# Electrical Conductance Mechanism of Silver–Polyacrylonitrile Nanocomposite Fibers

**DOI:** 10.3390/ma16083085

**Published:** 2023-04-13

**Authors:** Olga Rac-Rumijowska, Helena Teterycz

**Affiliations:** Faculty of Microsystem Electronics and Photonics, Wroclaw University of Science and Technology, Janiszewskiego 11/17, 50-372 Wroclaw, Poland

**Keywords:** polyacrylonitrile, silver nanoparticles, nanocomposite fibers, conductive fibers

## Abstract

This paper presents the mechanism of electrical conductivity in nanocomposite polyacrylonitrile (PAN) fibers modified with silver nanoparticles (AgNPs). Fibers were formed by the wet-spinning method. The nanoparticles were introduced into the polymer matrix as a result of direct synthesis in the spinning solution from which the fibers were obtained, thereby influencing the chemical and physical properties of the polymer matrix. The structure of the nanocomposite fibers was determined using SEM, TEM, and XRD, and the electrical properties were determined using the DC and AC methods. The conductivity of the fibers was electronic and based on the percolation theory with tunneling through the polymer phase. This article describes in detail the influence of individual fiber parameters on the final electrical conductivity of the PAN/AgNPs composite and presents the mechanism of conductivity.

## 1. Introduction

Typical polymers are dielectrics with a resistivity of 10^9^–10^20^ Ω·cm. For this reason, electronic devices are mainly used for the production of electronic housings or insulating elements. The change in the perception of polymers was initiated by the synthesis of conducting polymers [1] and the preparation of conductive polymer composites. Since then, the use of polymers and plastics in electronics has attracted more and more interest, especially in connection with the development of flexible and wearable electronics. Compared to the traditional technology based on silicon substrates, polymers enable the construction of light, cheap, and flexible electronic components [2]. Conductive polymer composites are of particular interest. As the conductive phase in such composites, the following materials are most often used: silver [3], carbon black [4], or graphene [5]. These materials find many applications in electronics, and the most popular ones include, above all, making conductive, flexible paths and other electronic components on flexible polymer films [3] and, recently, on fabrics [6]. Thanks to this, the development of electronic textiles or E-textiles has been observed.

The conductivity mechanism of conductive composites is based on a combination of two mechanisms: percolation [7], involving the creation of a conductive filler path, and tunneling [8] through a thin, dielectric polymer phase surrounding conductive particles. However, in many statistical models [9] and publications [10] on conductive composites, the effect of tunneling is neglected, even though its influence on the composite conductivity is very important even after exceeding the percolation threshold, because, even then, there is no real physical contact between adjacent filler particles, and conduction occurs by tunneling between the thin layers of polymer surrounding the filler particles when they are close to each other. The thickness of the boundary layer, allowing for charge tunneling, is in the range of 1–9 nm and depends mainly on the intensity of interactions between the polymer and the conductive phase. For this reason, it is thought that too high of a tunneling resistance should not be a factor limiting the conductivity of composites [11]. Bauhofer and Kovacs [12] found that the tunneling of electric charge carriers through boundary polymer layers has a dominant effect on the overall conductivity of the composite. In most real systems, the value of the percolation threshold deviates significantly from the values determined for ideal systems based on the statistical percolation theory. However, the occurrence of both these phenomena at the same time makes it often impossible to separate them completely and precisely determine the impact of these mechanisms on the value of the percolation threshold and the final value of the electrical conductivity of the composite [13].

The use of nano- instead of microparticles of conductive filler in composites improves the mechanical parameters of the composite and should significantly improve its electrical conductivity. In the case of using nanomaterials, the percolation threshold, i.e., the formation of the first conductive path in the composite, followed by a rapid increase in electrical conductivity, occurs with a much smaller amount of conductive filler than when using bulk particles [14]. However, the use of nanomaterials is associated with various problems, including their high tendency to agglomerate. For this reason, the introduction of nanoparticles into the matrix material causes the formation of a large number of defects in the structure of the material. In this case, the real properties of the nanocomposite differ significantly from those predicted theoretically. The weakening of the strength of the material is often observed. This is because the parameter determining the properties of the nanocomposite is the degree of dispersion of the filler in the matrix, which is particularly influenced by the morphology of the nanoparticles and their distribution in the composite. The agglomeration tendency of nanomaterials is their natural feature resulting from a very large specific surface area and, thus, from the high surface energy of nanoparticles; accordingly, the precise dispersion of dry nanoparticles in the polymer matrix is energy-intensive and practically impossible [15]. Schaefer and Justice [16], on the basis of an extensive analysis of the literature on the preparation of nanocomposites, showed that the use of nanoparticles as fillers in composites usually does not lead to the formation of nanocomposites. Almost without exception, numerous micron structures are present in these composites, regardless of the shape and type of filler used. For the above reasons, despite the high availability of various types of nanomaterials, the preparation of real nanocomposites is still a major technological and scientific challenge.

Difficulties in the uniform dispersion of the filler are particularly significant in the case of the production of composites in the form of fibers or yarns. Agglomeration of the dispersed phase in bulky composites causes the weakening of their mechanical strength; however, in the case of fibers with a diameter slightly larger than the size of the agglomerate, the presence of agglomerates destroys the fiber structure. The problem of filler dispersion in nanocomposites is extremely important. For this reason, various proposals for solving problems related to the dispersion of nanoparticles are slowly being developed so that their distribution in the polymer matrix is homogeneous. Fakirov [17] presented the concept of converting instead of adding, in which he indicates the possibility of producing a polymer nanocomposite with perfect dispersion of the nanomaterial in the matrix.

The presented concept of obtaining nanocomposite polyacrylonitrile fibers as a result of in situ synthesis of nanoparticles in a spinning solution fits perfectly into the concept presented above and prevents the formation of agglomerates. In our previous articles, we presented the method of obtaining nanocomposite fibers [18] and discussed in detail the influence of nanoparticles on the structural parameters of fibers [19]. In this work, the effect of silver nanoparticles on the electrical conductivity of fibers and the proposed mechanism of this conductivity are presented. This mechanism is partly based on the mechanism proposed to describe the electrical conductivity of glasses.

The obtained fibers were characterized by a resistivity of 10^7^ to 10^9^ Ω·cm. In the literature, one can find numerous publications on the preparation of composite PAN fibers modified with silver nanoparticles [20,21,22,23], including those with electrical parameters [24,25] similar to those in the presented paper. However, most of the publications concern the electrospinning process, in which the polymer solution is characterized by a much lower viscosity, whereby the dispersion of nanoparticles does not cause such difficulties as in the wet spinning method. This method, unlike electrospinning, where nanofibers are obtained, enables the production of fibers used in the textile industry [26].

## 2. Materials and Methods

### 2.1. Materials

Dimethylformamide (DMF) from POCH Polska was used as a solvent for the fiber-forming polymer—polyacrylonitrile (PAN). As a stabilizing agent, polyvinylpyrrolidone (PVP) by Sigma-Aldrich (Schnelldorf, Germany) was used, which was dissolved in DMF, obtaining a solution with a concentration of 111 g/dm^3^ (PVP), which corresponds to a solution with a concentration of 1 mol/dm^3^ in terms of the molar mass per liter. The precursor of Ag^+^ ions was a 1 M aqueous solution of silver nitrate (V) (AgNO_3_). A 0.2 M solution of ascorbic acid (C_6_H_8_O_6_) made in DMF was used as a reducing agent. All solutions were prepared immediately prior to fiber preparation.

### 2.2. Spinning of Fibers

Nanocomposite PAN fibers were obtained as a result of the direct synthesis of silver nanoparticles in a spinning solution. Our earlier studies described the method of obtaining silver nanoparticles in a polyacrylonitrile solution [18], as well as the method of obtaining and structural characterization of nanocomposite fibers [19]. In this study, the fibers were obtained using the same method, according to a Polish patent [27]. The silver content in the fibers ranged from 1% to 3% by weight.

The fibers were formed by wet spinning. The spinning solution contained 23% PAN in DMF and was obtained by stirring at 40–43 °C. PVP was added to the finished spinning solution in a molar ratio to the amount of silver ions of 4:1, followed by silver nitrate in the amount of 1%, 2%, or 3% by weight of silver in relation to the weight of the finished fibers and ascorbic acid in a molar ratio to the amount of silver ions of 1:1. After adding the reducing agent, the spinning solution was stirred for 2.5 h at constant temperature.

In the fiber spinning process, the spinning solution was forced through a 240-hole nozzle and immersed in two baths: (1) a solidifying bath containing 60% DMF in water at 20 °C, and (2) a plasticizing bath containing 50% DMF in water at 70 °C. In both baths, the solvent and possible dirt from the fiber volume were removed. According to an analysis of the baths by UV/Vis spectrometry, no silver was found in any of them [18]. Thus, silver nanoparticles were not leached from the fiber volume in any of the baths, which proves their good fixation in the polymer volume and the complete transition of silver from the spinning solution to the fibers. On this basis, the authors assumed the added amount of silver as the amount present in the fibers.

### 2.3. Characterization

We performed the characterization of fibers by high-resolution transmission electron microscopy (HRTEM) FEI Tecnai G2 20 XTWIN (FEI, Inc., Valley City, ND, USA). The cross-section of the fibers was observed and obtained using a high-resolution SEM Xe-PFIB FEI Helios (Bruker Corporation, Billerica, MA, USA). A single fiber was covered with a platinum layer, and then a cross-section was made with a focus ion beam (FIB). The crystallographic structure of the obtained fibers was studied using a Philips Materials Research Diffractometer (Philips, Amsterdam, The Netherlands) with CuKa radiation. The θ/2θ scan, typical of powder materials, was used.

The study of electrical conductance was performed along the fibers. Before the measurements, the fibers were stored in an atmosphere with a relative humidity of 60%, at a temperature of 20 °C for 24 h. The tests were carried out at a constant temperature of 20 °C, in an ambient atmosphere with a relative humidity of 30%. AC tests were performed using the impedance spectroscopy (SI) method with the Solartron 1260 analyzer (West Sussex, UK). Measurements were made in the range from 1 Hz to 1 MHz with 121 points per decade, using a sinusoidal voltage signal with an amplitude of 2 V. DC tests were performed using the Keithley Instruments 610C Solid-State Electrometer device (Cleveland, OH, USA).

Typically, the resistivity of any material is determined using Equation (1), based on the knowledge of the resistance of a sample of known dimensions.

(1) $$\rho =R\frac{A}{l},$$

where *R* is the sample resistance, *A* is the cross-sectional area of the sample, and *l* is the sample length.

However, in the case of yarns consisting of several hundred fibers with a diameter of several micrometers, it is practically impossible to specify the cross-sectional area. For this reason, textile yarns are characterized by another parameter called mass-specific resistance *R_s_*, which better reflects the electrical properties of a material with such fibers [28]. The mass-specific resistance is described by Equation (2); its unit is Ω·g/cm^2^, and it is commonly used in the literature to determine the electrical parameters of fibers.

(2) $$R_{s}=\frac{RNm_{l}}{l\cdot 10^{5}},$$

where *N* is the number of filaments in the yarn, *m_l_* is the linear mass [tex] = [g/km], and *l* is the sample length (cm).

**Figure 1:**
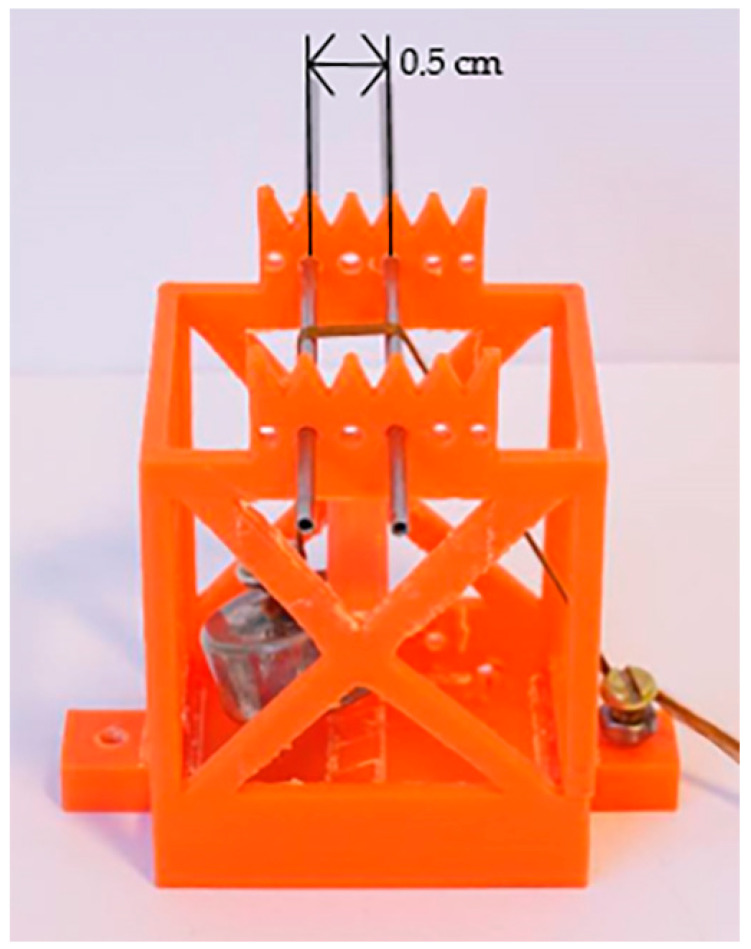
System for electrical characterization of fibers.

To obtain reliable electrical contacts, a two-electrode measuring system with a construction similar to that proposed in the literature was made [29]. This holder ensured electrical contact of the electrodes with the maximum number of fibers (Figure 1). Suspension of the weight of appropriate mass at the end of the fiber enabled the improvement of electrical contact. Here, 50 g was taken as the optimal value.

## 3. Results

**Figure 2:**
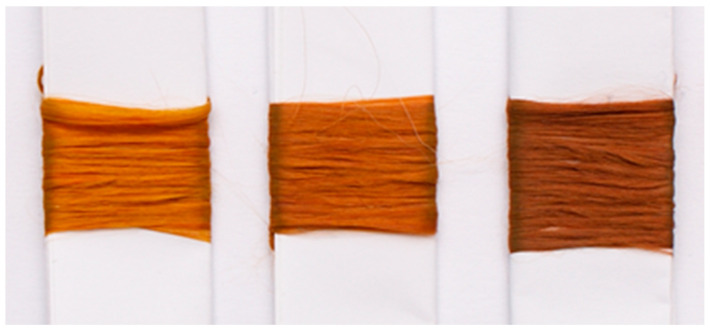
Photography of PAN fibers with silver content by weight (from the left): 1%, 2%, and 3%.

As a result of the modification of PAN fibers with silver nanoparticles, their color changed from white (unmodified PAN fibers) to orange-brown, with the intensity dependent on the concentration of nanoparticles (Figure 2). The characteristic color of nanocomposite fibers is the result of plasmon resonance occurring as a result of the interaction of electromagnetic radiation with noble metal nanoparticles [30]. Under resonance conditions, a strictly defined amount of incident light energy is absorbed by the plasmons. This phenomenon occurs for particles whose diameter is much smaller than the wavelength of the incident light. The absorption peak for spherical silver nanoparticles is in the radiation range of 400–450 nm depending on the size. In our previous study [19] using UV-Vis tests, we demonstrated the presence of silver nanoparticles in the fibers. Their presence was evidenced by a characteristic peak around 420 nm.

**Figure 3:**
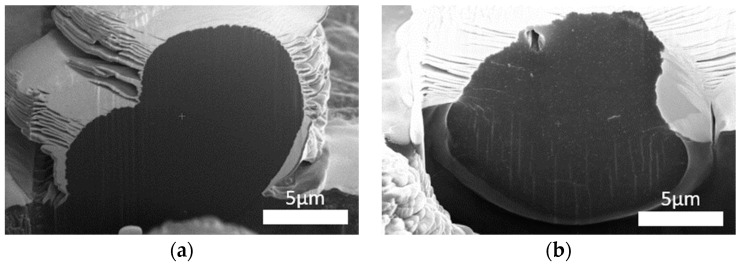
Fiber cross-sections (SEM images): (**a**) non-doped; (**b**) containing AgNPs.

The silver nanoparticle distribution in the fibers was determined through microscopic observation. FIB/SEM imaging was performed for the cross-sections and longitudinal sections of the fibers. In addition, an HRTEM analysis of the polymer lamella was performed. The observations revealed that the cross-sections of undoped fibers and those containing silver nanoparticles did not differ (Figure 3). In both cases, the structure was smooth and did not contain cracks or imperfections.

**Figure 4:**
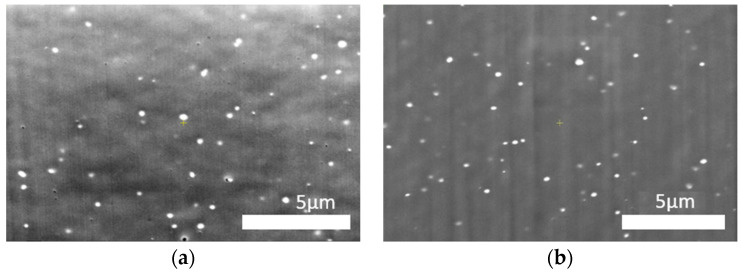
Distribution of nanoparticles in PAN fibers: (**a**) cross-section; (**b**) longitudinal section.

SEM imaging, performed in the material contrast mode, showed that silver nanoparticles were distributed uniformly in both sections of the fiber (Figure 4).

**Figure 5:**
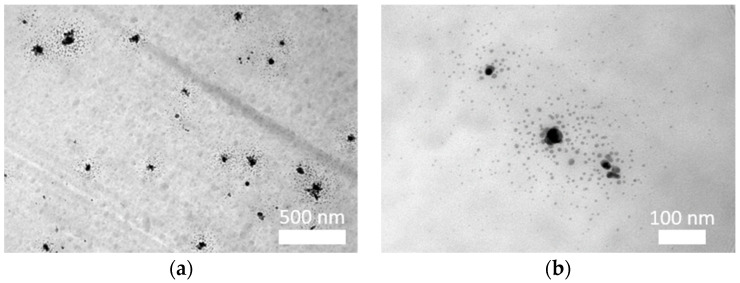
HRTEM images of the distribution of nanoparticles in PAN fibers: (**a**) cross-section; (**b**) longitudinal section.

HRTEM microscopic analysis showed that the silver nanoparticles were distributed in the form of clusters consisting of particles with a diameter of about 20 nm (Figure 5). These particles were surrounded by a large number of smaller nanoparticles with a diameter of several nanometers, which is consistent with the previously published results of research performed using the DLS method [18]. A greater concentration of particles in certain areas of fibers may result from the location of nanoparticles primarily in amorphous areas of fibers.

Our previous research [19] showed that the addition of silver nanoparticles in the amount of 1 wt.% increased the mechanical tensile strength of the fibers by 6% and Young’s modulus by as much as 46%. This was related to the good dispersion of nanoparticles in the fiber volume.

### 3.1. XRD

**Figure 6:**
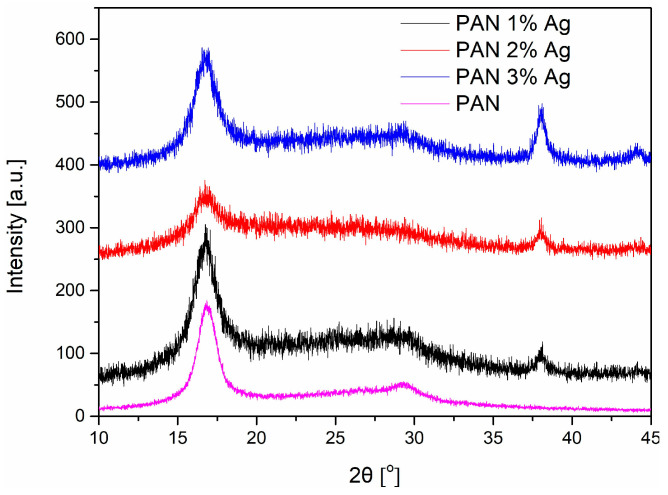
X-ray diffractograms of polyacrylonitrile fibers with different silver content.

The fiber crystal structure was determined through XRD studies. The diffractograms of polyacrylonitrile fibers showed two characteristic peaks at 17° and 29° (Figure 6). The intense peak at the 2θ angle of 17° was characteristic of the (001) plane of PAN crystallites with a hexagonal structure. The diffractograms of all silver-modified fibers clearly showed a peak at the 2θ angle of about 38°, characteristic of silver’s crystallographic plane (111).

According to the XRD results and the Hinrichen method (Equation (3)), the degree of crystallinity of the fibers was determined. Since some peaks were difficult to separate, the diffraction curve was analyzed by creating the theoretical function with the best fit to the experimental curve. The curve fitting was performed using the Rosenbrock method. This method enabled a good fit in WAXSFIT [31].

(3) $$X_{c}=\left[\frac{I_{c}}{I_{c}+I_{a}}\right],$$

where *I_c_* is the integral under the peaks corresponding to the crystalline phase of the polymer, and *I_a_* is the integral under the peaks corresponding to the amorphous phase of the polymer.

Using the Debye–Scherrer equation (Equation (4)), the average crystallite size of the PAN fibers was calculated on the basis of the half-width of the peak at about 17° (Table 1). On the other hand, the average size of silver nanoparticle crystallites was determined on the basis of the half-width of the peak occurring at about 38° (characteristic for the crystallographic plane (111) of silver). The presence of silver nanoparticles increased the degree of crystallinity from 0.33 to 0.47, regardless of the amount of silver addition. The size of silver crystallites increased with the increase in silver content in the fiber and ranged from 7 to 11 nm.

(4) $$L_{\left(hlk\right)}=\frac{K\cdot \lambda }{\beta \cdot cos\theta_{max}},$$

where *L_(hlk)_* is the average size of crystallites, *K* is the Scherrer constant (0.89), *θ_max_* is the angle for the maximum peak [rad], *λ* is the length of the radiation beam 1.542 [Å], and *β* is the half-width of the peak [rad].

### 3.2. Electrical Conductivity

**Figure 7:**
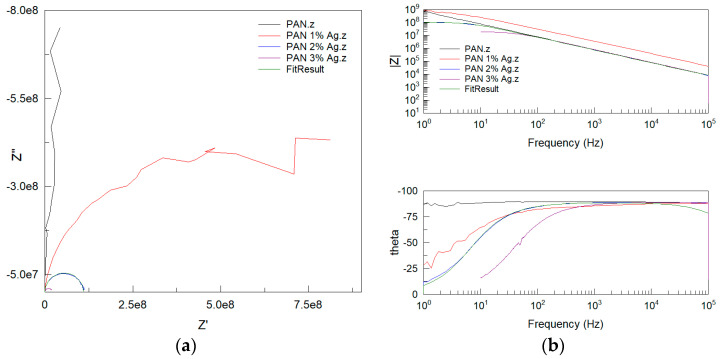
Nyquist diagram of fibers. Nyquist (**a**) and Bode (**b**) plots of fibers. The fitting result is shown for the PAN 2% Ag fiber.

**AC testing.** Analysis of the impedance spectroscopy measurements showed that the fibers’ electrical parameters changed upon introducing silver nanoparticles into them (Figure 7).

**Figure 8:**
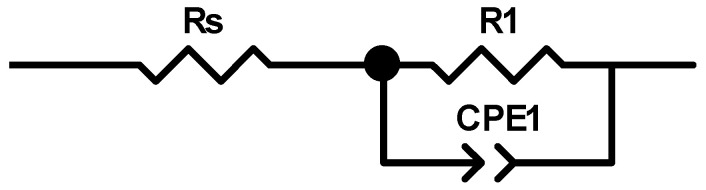
Equivalent Circuit Model.

An electric equivalent model (Figure 8) was proposed, consisting of three elements:

- Resistance of electrical connections (Rs);
- Linear resistance of the sample (R1);
- Constant phase element (CPE).

This model contained two components: the capacitance of the capacitor (CPE-T), and the so-called “capacitor ideal” (CPE-P). The closer the value of this parameter is to 1, the more the element is a lossless capacitor. The parameters of the individual elements of the equivalent model of the system are given in Table 2. The linear resistance of the unmodified polyacrylonitrile fibers was 1.6 · 10^11^ Ω/cm, which is characteristic for dielectric materials. Nanocomposite fibers containing silver had a much lower linear resistance at the level of materials with potential electrostatic applications. The concertation of 1 wt.% Ag changed the linear resistance to two orders of magnitude lower than the reference fibers, and the addition of each additional percentage of silver reduced the linear resistance by another order of magnitude. The fiber containing 3 wt.% Ag had a linear resistance of 1.9 · 10^7^ Ω/cm, indicating that the material was already at the same level of conductivity as semiconductors.

**DC testing**. On the basis of the current–voltage characteristics of PAN fibers containing silver nanoparticles, the resistance of the fibers was determined; using Equation (2), the mass-specific resistance as a function of voltage was determined.

**Figure 9:**
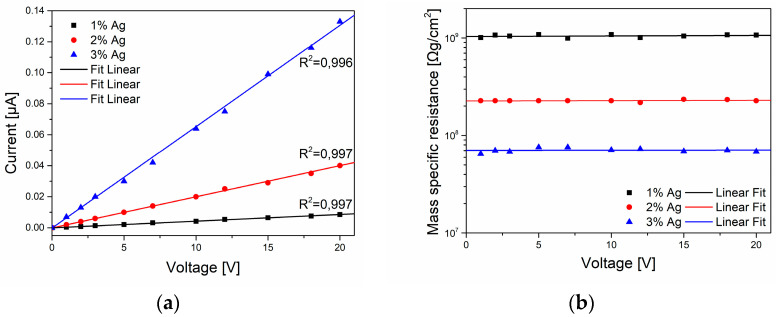

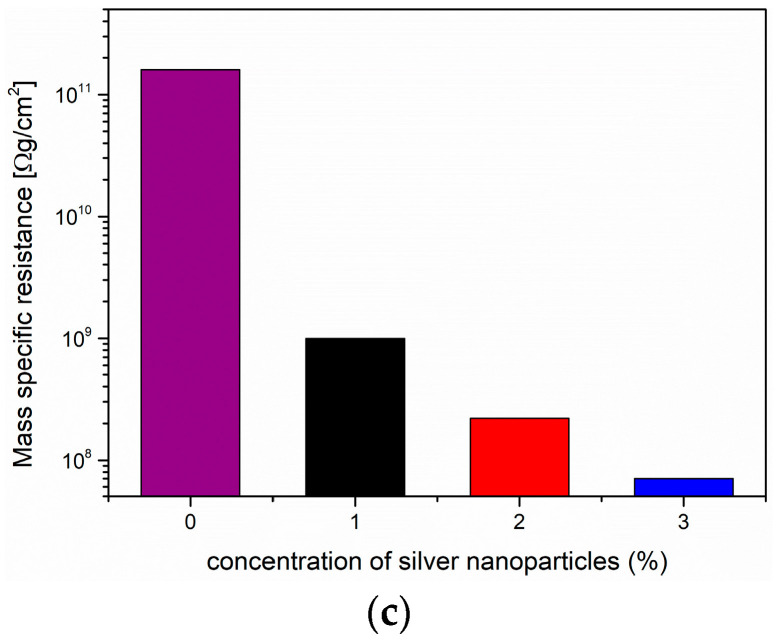
Electric parameters of PAN fibers as a function of the silver nanoparticle concentration: (**a**) current–voltage characteristics; (**b**) change in mass resistance as a function of voltage, (**c**) mass resistance as a function of silver concentration.

The results analysis showed that the current value changed linearly with the increase in voltage (Figure 9a) and depended on the concentration of silver. The linear relationship proved the electronic conductivity in nanocomposite fibers. Mass-specific resistance practically did not change as a function of voltage (Figure 9b). However, its clear decrease was observed as a result of fiber modification with nanoparticles, which significantly depended on the silver content in the fibers (Figure 9c). Importantly, the presented data are consistent with the results obtained using the AC method (Table 2).

## 4. Discussion

The classic conductivity mechanism of polymer composites is based on the percolation theory, according to which the change in electrical properties from dielectric to highly conductive is associated with the formation of conductive paths through the conductive filler in the polymer matrix. The point where there is a step change in the electrical properties is called the percolation threshold. However, exceeding the percolation threshold in many cases does not result in very high electrical conductivity. This was exactly the situation in this study (Table 2). The linear resistance of the unmodified polyacrylonitrile fibers was 1.6 · 10^11^ Ω/cm (dielectric materials); for the fiber containing 3 wt.% Ag, after the percolation threshold, the linear resistance was 1.9 · 10^7^ Ω/cm (level of semiconductors). According to the literature data, the maximum conductivity of the composite that can be obtained, despite exceeding the percolation threshold, is about 2–4 orders lower than the conductivity of the conductive filler used [11,31]. This is due to the tunneling of the charge through a thin layer of polymer between the particles of the conductive filler. Therefore, in such a system, it is extremely important to take into account the properties of the polymer matrix.

**Figure 10:**
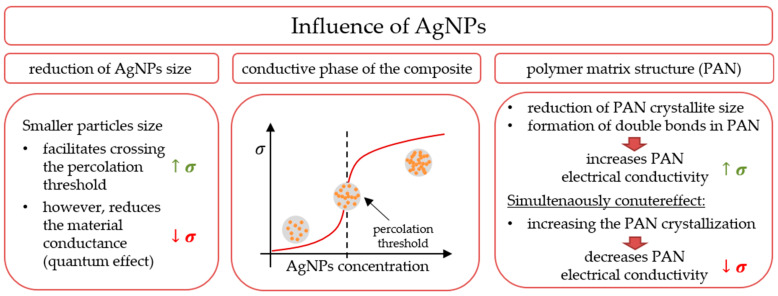
Impact of silver nanoparticles on electrical conductivity and structure of nanocomposite PAN/Ag fibers (σ—conductivity of material, ↑ and ↓—increase and decrease).

Polyacrylonitrile fibers were doped with silver nanoparticles synthesized in situ in the spinning solution. As a result of direct nanoparticle synthesis, many chemical and physical interactions were created between the two materials during the synthesis and spinning process. These influenced the chemical and structural parameters of the polymer matrix. Therefore, when analyzing the mechanism of electrical conductivity of such fibers, in addition to considering the percolation theory and the particle size, one should also take into account the aspect related to the changes occurring in the polymer matrix under the influence of silver nanoparticles. The final electrical properties of the composite are influenced by the structure of the polymer matrix, the size of nanoparticles, and percolation effects (Figure 10), whereby all these phenomena occur simultaneously, and it is not possible to analyze their influence on each other.

### 4.1. Dependence of Electrical Conductivity on the Polymer Matrix Structure

Polyacrylonitrile, like most polymers, is a dielectric. According to the literature [28,29] and our measurements (Table 2), the conductivity of pure PAN at room temperature is about 1.5 · 10^–11^ S·cm^−1^. XRD studies (Figure 6) showed that PAN fibers have a semi-crystalline structure, and silver nanoparticles cause a significant change in their crystalline structure, which affects electrical conductivity. The degree of matrix crystallinity increased by 42% compared to unmodified fibers, and the size of PAN crystallites slightly decreased (Table 1). This change had a positive effect on the mechanical properties of the fibers, as presented in our previous work [19]. However, an increase in the degree of crystallinity causes a decrease in the electrical conductivity of the polymer. According to the literature, in the case of polyester fibers, an increase in the degree of crystallinity by 30% resulted in an increase in resistivity by three orders of magnitude [32]. The electrical conductivity of the fibers also depends on the average size of the crystallites. A smaller size of the crystallites (as observed in the obtained composite fibers) facilitates the transport of electric charges in the semi-crystalline material. To sum up, both changes in the crystalline structure of the polymer under the influence of silver nanoparticles have the opposite effect on the electrical conductivity of the matrix.

In addition to the physical structure of the polymer, its chemical structure affects the electrical conductivity. In nanocomposite fibers, the presence of nanoparticles favors the dehydrogenation process, as a result of which C=C double bonds appear in the polyacrylonitrile chain (according to FTIR studies in our previous study [19]). The intensity of the dehydrogenation process increases with the increase in the concentration of nanoparticles in the fibers. A similar system of conjugated double bonds occurs in electronically conductive polymers that have a linear structure and do not form lamellar crystalline regions [33]. In the case of the studied nanocomposite fibers, double bonds are formed as a result of the interaction of PAN nitrile groups with silver nanoparticles located in amorphous areas. As a result, these bonds are also formed in the amorphous region.

Thus, in nanocomposite fibers, in addition to the conductivity caused by the presence of silver nanoparticles, there is a small electronic conductivity of the amorphous polymer phase caused by the presence of a system of conjugated double bonds. The occurrence of this phenomenon, therefore, increases the conductivity of the amorphous phase of the polymer and facilitates the tunneling process.

### 4.2. Influence of Silver Nanoparticle Size

When analyzing the percolation phenomena in the nanocomposite fiber, it should be noted that the conductivity value depends not only on the properties of the polymer but also on the size, concentration, and shape of the filler particles. Increasing the concentration of silver nanoparticles in the fibers to 3% by weight resulted in a decrease in mass-specific resistance by almost four orders of magnitude (Figure 9c). The change is large, but still not what you would expect from a conductive composite. The reason for this, in addition to the tunneling phenomenon [12], is the size of the particles. The use of nanoparticles makes it possible to significantly reduce the required filler volume necessary to exceed the percolation threshold [34]. According to Xue [35], when the particle size of the conductive filler is about 200 nm, the percolation threshold occurs at a particle content of about 5% by volume. Further reducing the size of the conductive filler makes it possible to exceed the percolation threshold with even lower concentrations. However, when the size of metallic nanoparticles decreases, their conductivity also decreases. This effect is caused by a change in the crystal structure in the near-surface area and by quantum effects.

The subsurface area of nanomaterials is remodeled and becomes more disordered [29]. It is commonly assumed that nanoparticles have a core–shell structure, where the core has a crystalline structure, and the surface shell has an amorphous structure [14]. For this reason, they are characterized by lower conductivity.

In addition, in bulk metal crystals, electrons can move in all directions, and the mobility of electrons (*μ*) determines the conductivity and is described by Equation (5).

(5) $$\sigma \left(\mu \right)=\mu n\left|ze\right|,$$

where *μ* is the ion mobility, *n* is the ion concentration, *z* is the ion oxidation state, and *e* is the electron charge.

The electron mobility in bulk materials decreases mainly as a result of phonon scattering, scattering on structural defects or interfacial boundaries, and this is what the mean free path of electrons *λ* depends on. When the particle size is on the order of nanometers, changes occur in the electronic structure of the material. At enough small metal particles, discrete energy levels begin to dominate, and Ohm’s law breaks down. If the size of the particles is smaller than the mean free path of the electron (*λ*), then the electron mobility decreases as a result of additional electron scattering on the nanoparticle surface [36]. If the diameter of silver nanoparticles is smaller than 54 nm, the free path of the electron changes, as a result of which the electron mobility and, thus, the conductivity of the nanoparticles decrease [37]. In this case, the change in conductivity is described by the relationship [38] in Equation (6).

(6) $$\mu =\frac{e\lambda }{4\pi \varepsilon_{0}m_{e}V_{F}},\;$$

where *λ* is the mean free path of the electron, *m_e_* is the effective mass of the electron, *V_F_* is the electron velocity, and *ε*_0_ is the dielectric constant in a vacuum.

Considering the above, the obtained silver nanoparticles were characterized by a lower conductivity than bulk silver (*σ* Ag = 6.1 · 10^5^ S/cm); hence, obtaining polyacrylonitrile fibers with a resistance of 100 MΩ, containing 3 wt.% of silver is a very good result. For comparison, the calculated value of the resistance of a silver wire with a diameter of 5 nm and a length of 1 cm (analogous to a single percolation path made of nanoparticles with a diameter of 5 nm) is on the order of 10 MΩ. The resistance of the nanowire was calculated assuming a constant value of silver conductivity, independent of its diameter; as previously mentioned, the conductivity of materials decreases after exceeding their limit size.

### 4.3. Electrical Conductivity of Nanocomposite Fibers

The electrical conductivity of materials can be ionic, electronic, or mixed. Most of the proposed mechanisms of electrical conductivity of natural fibers are related to ionic conductivity, but there are also few reports indicating electronic conductivity [39]. The ionic nature of the conductivity of textile fibers is evidenced by the polarization occurring during tests conducted in an atmosphere with increased humidity. However, this may be due to the absorption of moisture by natural fibers. The change in the electrical conductivity of the fibers as a function of temperature and humidity can be explained in two ways. According to the first, the change in the electrical conductivity of the fibers is caused by a change in the concentration of electric charge carriers, i.e., ions, under the influence of humidity. According to the second, it is believed that the mobility of ions changes or polarization of charges takes place, which changes under the influence of an external electric field [40].

In the case of other amorphous dielectrics, such as glasses, electrical conductivity is described by, among others, the weak electrolyte model, in which it is assumed that the concentration of mobile charge carriers (ions) is small and the conductivity of the material mainly depends on it, according to Equation (7).

(7) $$\sigma \left(n\right)=\mu n\left|ze\right|,$$

where *μ* is the ion mobility, *n* is the ion concentration, *z* is the ion oxidation state, and *e* is the electron charge.

A model that is also used to describe the conductivity of glasses is the cluster avoidance model, in which charge carriers are assumed to move through amorphous regions surrounding more ordered regions.

**Figure 11:**
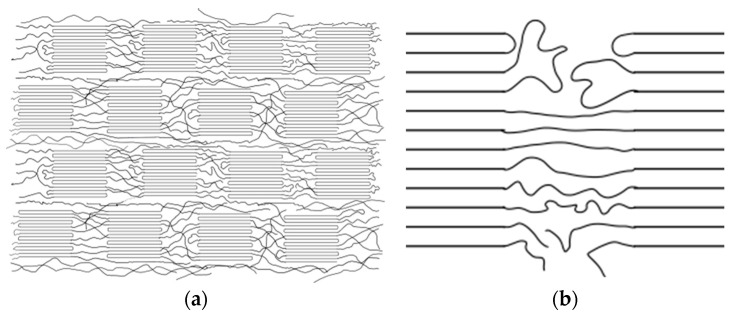
Scheme: (**a**) semicrystalline structure of polymer fibers; (**b**) amorphous area of the fiber.

Taking into account the semi-crystalline structure of PAN fibers, in which the crystallites are arranged in the amorphous phase along the fiber axis (Figure 11a) and the fact that the crystalline areas are characterized by higher resistance than the amorphous areas [41], for the description of the transport of electric charges in the tested polyacrylonitrile nanocomposite, a more adequate model is that of bypassing crystallites, especially considering that the admixture (silver) used contains extensive electric charge carriers. The greater conductivity of amorphous areas is associated with a much lower order of polymer chains (Figure 11b) and a greater number of defects. In conductive polymers, the transport of electric charges also depends on the concentration of defects occurring in polymer chains, such as chain ends, their entanglements, and structural defects [42]. Higher conductivity is observed in conductive polymers with low chain orientation [43]. The reason for such behavior of polymer crystallites (areas of high order) is their compact structure, which does not contain free electric charge carriers, and the fact that they are difficult to polarize.

**Figure 12:**
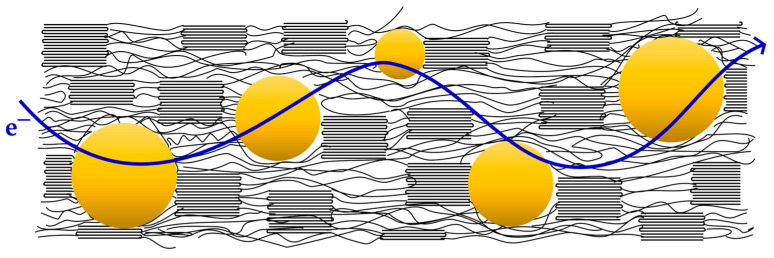
Conductivity scheme of nanocomposite polyacrylonitrile fibers.

According to the literature data presented above and the results of our research, the authors propose a mechanism of electrical conductivity of PAN/AgNPs nanocomposite fibers similar to the crystallite bypass mechanism. In the case of PAN/AgNPs fibers, the authors believe that the conductivity is mainly mediated by metallic silver nanoparticles (which results from the formation of a percolating path) located in the amorphous regions of the polymer, and as a result of tunneling occurring in the amorphous phase of polyacrylonitrile containing double bonds (Figure 12). In addition, taking into account the results of the tests presented on the current–voltage characteristics (Figure 9a), it should be stated that this conductivity is electronic. The proposed mechanism was presented in the author’s doctoral dissertation [44].

## 5. Conclusions

In conclusion, on the basis of the literature data and the results of our research presented in the article, it should be stated that silver nanoparticles in the obtained nanocomposite fibers affect the structural and chemical parameters of the polymer matrix, as well as increase the conductivity of the composite according to the percolation theory.

At the same time, the change in the structure of the polymer also has a significant impact on its electrical properties, because silver nanoparticles cause an increase in the degree of crystallinity of the matrix, thereby reducing conductivity, a decrease in the average crystallite size, thereby increasing conductivity, and the formation of C=C double bonds in the PAN chain, contributing to an increase in conductivity.

The linear resistance of standard polyacrylonitrile fibers is about 10^11^ Ω/cm, and that of the obtained nanocomposite fibers containing silver nanoparticles synthesized in situ ranges from about 5 · 10^7^ to 1 · 10^9^ Ω/cm, depending on the content of silver nanoparticles—1, 2, or 3 wt.% Ag. The addition of 3% Ag caused the formation of a percolating path and a significant decrease in the resistance of the fibers to the level of materials with electrostatic applications. The obtained conductivity results of the PAN/Ag material are similar to the results for fibers with the same silver content, but made by electrospinning, as presented in [24,25].

This paper described the influence of silver nanoparticles on the electrical conductivity of fibers and proposed a model describing the nature of conductivity, defined as a crystallite avoidance model based on the theory of percolation, taking into account the phenomenon of tunneling through the polymer phase. This work is the third publication in the triptych, along with [18,19], concerning the full description of the preparation and characterization of nanocomposite polyacrylonitrile fibers doped in situ with silver nanoparticles.

## 6. Patents

Fibers were obtained using a method protected by a Polish patent “Method for preparing modified polyacrylonitrile fibers”, PL 220522, Poland 2015.

## Figures and Tables

**Table 1:** Crystallinity degree of nanocomposite PAN fibers and size of crystallites.

Fibers	Crystallinity Degree	L(001) [nm] PAN	L(111) [nm] Ag
PAN	0.33	6	--
PAN Ag 1%	0.47	5	7
PAN Ag 2%	0.47	4	10
PAN Ag 3%	0.47	4	11

**Table 2:** Values of particular elements of the equivalent circuit model.

Fibers	Rs [Ω]	R1 [Ω/cm]	CPE1-T [F]	CPE1-P
PAN	1520	1.6 × 10^11^	5.28 × 10^−11^	0.97
PAN Ag 1%	1520	6.3 × 10^9^	6.40 × 10^−11^	0.96
PAN Ag 2%	1520	1.3 × 10^8^	2.28 × 10^−11^	0.98
PAN Ag 3%	1520	1.9 × 10^7^	1.94 × 10^−10^	1.00

## Data Availability

Not applicable.
